# Clinical application and safety observation of levofloxacin in children with refractory Mycoplasma pneumoniae pneumonia

**DOI:** 10.1186/s12879-026-13433-0

**Published:** 2026-04-29

**Authors:** Tianhua Li, Lin Zhao, Xianliang Lang, Qian Li, Lexiang Yu

**Affiliations:** 1 Department of Paediatrics, Weifang People’s Hospital Affiliated to Shandong Second Medical University, 151 Guangwen Road, Weifang, Shandong 261041 China; 2 Department of Paediatrics, The Second People’s Hospital of Linqu County, Xinzhai Town, Linqu County, Weifang, Shandong 262610 China

**Keywords:** Levofloxacin, Refractory Mycoplasma pneumoniae pneumonia, Efficacy, Safety

## Abstract

**Background:**

In recent years, Mycoplasma pneumoniae (MP) has developed widespread resistance to macrolide antibiotics, and second-line anti-Mycoplasma drugs such as levofloxacin have begun to be used in children. However, there is currently insufficient data on their efficacy and safety.

**Objective:**

To explore the application effect of levofloxacin in children with refractory Mycoplasma pneumoniae pneumonia (RMPP) and evaluate its safety.

**Methods:**

This study is an exploratory research, selecting 82 RMPP patients treated with levofloxacin who were admitted to the Department of Pediatric Respiratory Medicine at Weifang People’s Hospital between November 2023 and December 2024 as the research subjects. Compared the blood routine and biochemical indicators before and after the application of levofloxacin, and observed the clinical efficacy and adverse reactions of levofloxacin.

**Results:**

The average fever reduction time after the application of levofloxacin was (1.96 ± 1.52) days. After the application of levofloxacin, there was no change in white blood cell (WBC), neutrophil (N), platelet (PLT) and other parameters in the blood routine (*P* > 0.05). The lymphocyte count (L) has increased but remains within the normal range. There was no difference in alanine aminotransferase (ALT) before and after treatment, but aspartate aminotransferase (AST) decreased compared to before. Lactate dehydrogenase (LDH) and creatine kinase isoenzyme (CKMB) decreased after medication (*P* < 0.05), while creatine kinase (CK) remained unchanged. Blood creatinine (CREA) and Blood urea (Urea) both decreased (*P* < 0.05), indicating no damage to liver and kidney function. There were a total of 9 adverse drug reactions related to levofloxacin, including 5 cases of rash, 2 cases of gastrointestinal reactions, 1 case of neurological reaction, and 1 case of lower limb pain. No other adverse reactions or sequelae were observed during the 12-month clinical follow-up.

**Conclusion:**

Levofloxacin has shown good efficacy in treating RMPP in children, with no serious adverse reactions detected in a short period of time.

**Supplementary information:**

The online version contains supplementary material available at 10.1186/s12879-026-13433-0.

## Background

In recent years, due to the widespread and inappropriate use of macrolide antibiotics, Mycoplasma pneumoniae (MP) has developed resistance to macrolide antibiotics. The resistance rate of MP varies among countries. Currently, the vast majority of MP isolates in East Asia, especially in China, are resistant to macrolide drugs, while in Europe and North America, their resistance rates are significantly lower than in Asia [1]. Jiang et al. [2] collected 4145 MPP respiratory samples from different regions of China between January 2013 and December 2019. After testing, it was found that the resistance rate of Mycoplasma pneumoniae pneumonia (MPP ) in China was very high, with Shanghai having the highest resistance rate at 100%, followed by Liaoning at 84.2%, Hunan at 83.3%, and Beijing at 75.8%. The drug resistance rate in children is higher than that in adults. The resistance mechanism of MP to macrolide antibiotics is mainly due to base mutations at positions 2063, 2064, or 2617 of the 23SrRNA gene. Among them, mutations at positions 2063 or 2064 can lead to high-level resistance, while base mutations at position 2617 can lead to low-level resistance [3]. MP infection faces enormous pressure in drug selection.

With the outbreak of MP infection in Chinese Mainland from 2023 to 2024, the incidence rate of RMPP remains high, which seriously threatens the health of children. Pediatricians have to use unapproved quinolone drugs to treat MP infections in children. The application of quinolones in the treatment of MP infection in children has not been reported abroad. In China, Shandong First Medical University Affiliated Provincial Hospital [4] and the Second Affiliated Hospital of Wenzhou Medical University [5] respectively reported 6 cases of RMPP treated with levofloxacin, but the clinical cases are relatively few. There is still a lack of large-scale research on its clinical efficacy, safety, and impact on children’s growth and development. The aim of this study is to investigate the therapeutic effect of levofloxacin on RMPP and evaluate its safety in pediatric use, providing important theoretical guidance for promoting its application in children.

## Methods

### Case selection

Children with MPP admitted to the Children’s Respiratory Ward of Weifang People’s Hospital in Shandong Province between November 2023 and December 2024 were selected as the study subjects. All patients were diagnosed with MP infection by respiratory pathogen nucleic acid testing and Mycoplasma antibody testing before or after admission. 82 children with RMPP treated with levofloxacin were selected as the experimental group for efficacy observation and safety analysis. Among them, there were 42 males and 40 females, with the youngest being 1 year and 8 months old and the oldest being 13 years and 10 months old. The average age was 6.97 ± 2.10 years old, and the average weight was 24.84 ± 8.67 Kg. All cases were lobar pneumonia, including 18 cases with pleural effusion and 5 cases with severe pneumonia. These 82 children were all treated with regular azithromycin or erythromycin for more than one week, and their condition was not controlled or worsened.

RMPP diagnostic criteria [6]:

1. MPP clinical diagnosis: Meets the diagnostic criteria for community-acquired pneumonia, and serological (MP IgM) and/or pathogen nucleic acid (MP-DNA) testing confirms Mycoplasma pneumoniae infection.
2. Refractory determination: After standardized use of macrolide antibiotics (such as azithromycin) for ≥ 7 days, at least one of the following conditions is still met: (1) persistent feve (body temperature>37.5℃). (2) The clinical symptoms continue to worsen and the pulmonary imaging progresses, such as the expansion of the lesion scope, increased lesion density or the appearance of new lesions, or the occurrence of atelectasis, pleural effusion, etc.

**Fig. 1:**
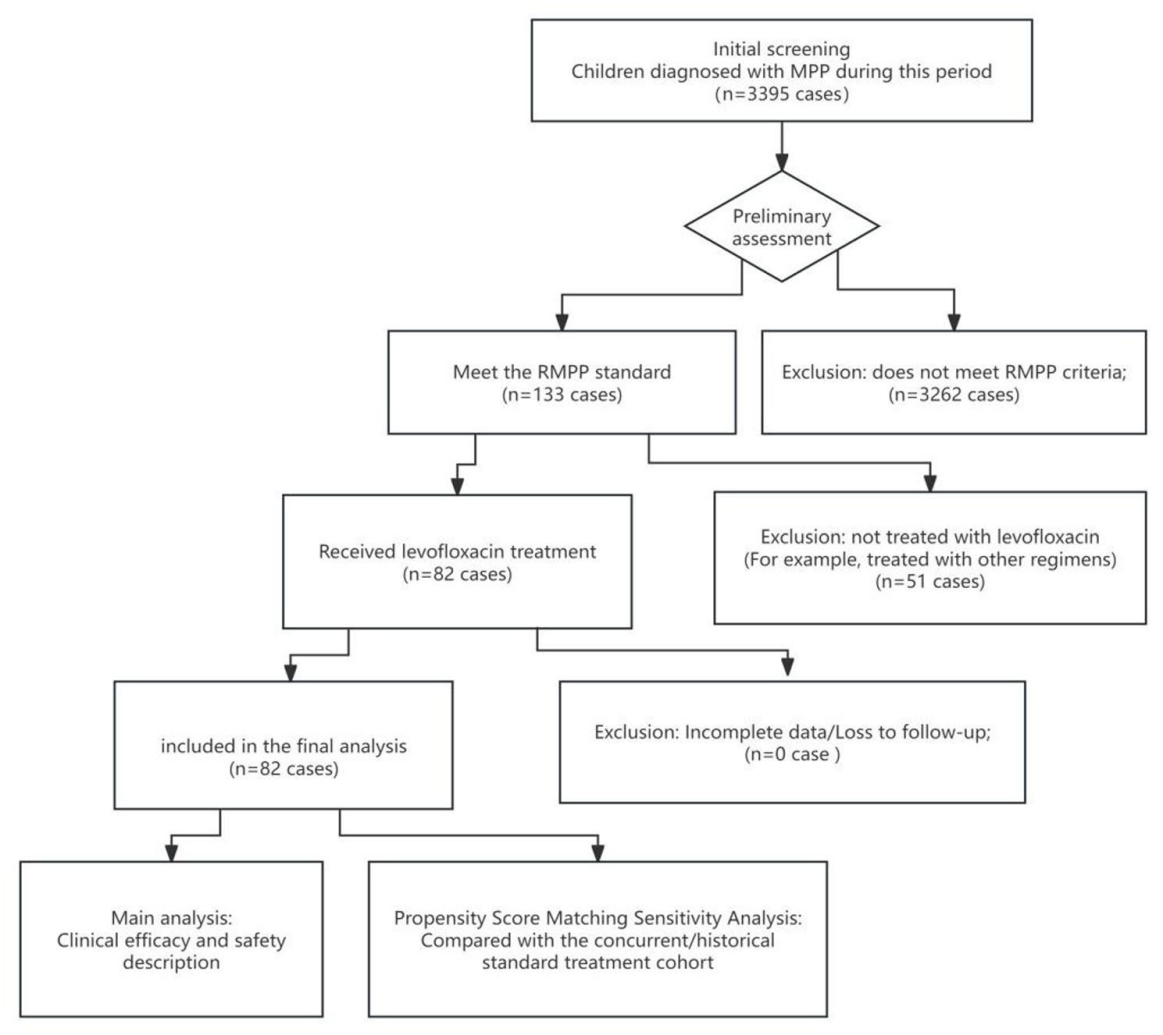
Patient flow diagram

Exclusion criteria: (1) Combination of adenovirus or other pathogenic bacterial infections. (2) Combination of thrombosis, embolism, or other important organ damage. (3) Incomplete information or missing data. (Patient flow diagram see Fig. 1)

The study was approved by the Ethics Committee of Weifang People’s Hospital (KYLL20240105-8). All procedures performed in this study were in accordance with the ethical standards of the Declaration of Helsinki (World Medical Association, 2024) [7], and all parents signed informed consent forms for their children’s medication beyond the instructions.

### Evaluation of macrolide resistance

This study evaluated the macrolide resistance of MP through the following two methods:

Molecular biology testing: With the voluntary consent of the patient’s guardian, real-time fluorescence quantitative PCR was used to detect the presence of A2063G and/or A2064G point mutations in the V region of the 23 S rRNA gene of MP in 35 children who retained qualified respiratory specimens (such as bronchoalveolar lavage fluid and throat swabs) before treatment, in order to confirm macrolide resistance. The drug resistance testing is completed by physicians from the Laboratory Department of Weifang People’s Hospital.

Clinical phenotype inference: Given the limited popularity of molecular testing in clinical practice, the determination of “refractory” (i.e. no response to standardized treatment of macrolides for ≥ 7 days) for children who have not undergone molecular testing can serve as indirect evidence of suspected macrolide resistance or abnormal host immune response in clinical practice.

### Levofloxacin dosing regimen

The dosage and usage of the medication were strictly in accordance with the “Diagnosis and Treatment Guidelines for Mycoplasma Pneumonia in Children (2023 Edition)” issued by the National Health Commission of China. 10 mg/(kg/time) was given to children aged 6 months to 5 years old, q12h; Children aged 5–16 years old, 10 mg/(kg/time), qd, Administer intravenous injection, with a maximum daily did not exceeding 400 mg, and switch to oral administration of the same dose after discharge. The treatment course generally did not exceed 10 days.

### Specimen collection and efficacy observation

All experimental group children were given fasting venous blood tests for blood routine and biochemical indicators before and after the application of levofloxacin, and underwent imaging examinations such as chest X-rays or lung CT scans. At the same time, observed and recorded the efficacy and adverse reactions of levofloxacin in the pediatric patients, and followed up for 12 months after discharge.

1. Complete cure criteria:

(1) No fever; (2) No clinical manifestations such as cough, sputum, wheezing, or pulmonary signs; (3) Complete absorption of pulmonary inflammation imaging.

2. CT evaluation time point: (1) Baseline (before treatment); (2) 7–10 days after the start of treatment (for severe cases or those with poor response); (3) At the end of this treatment/before discharge; (4) Outpatient follow-up 4–12 weeks after the end of treatment.

### Security assessment

All adverse events were actively collected and recorded according to a pre established monitoring plan, rather than retrospectively reviewing medical records. Using internationally recognized standardized terminology: We had systematically classified and coded adverse events using the “Medical Dictionary of Regulatory Activities”. We had adopted objective severity grading standards: we had standardized and quantified the severity of adverse events based on the “Common Adverse Event Evaluation Criteria” (1–5 levels). Unfortunately, not all children had undergone electrocardiogram examination, so a comprehensive evaluation of electrocardiogram, especially the detection of QTc, had not been carried out.

### Inspection method

All patients were tested on an empty stomach in the morning, and 5-10 ml of venous blood was drawn from the elbow vein or femoral vein by researchers. At the same time, throat swab specimens for respiratory pathogen nucleic acid were collected. All collected specimens were sent to the hospital’s laboratory department for testing by professional personnel.

### Statistical methods

This study used SPSS25.0 software (IBM, Armonk, NY, The experimental data in the United States were analyzed and processed, and *P* < 0.05 was considered statistically significant. Measurement data is represented by $\left( {\bar {x}\, \pm \,{\mathrm{s}}} \right)$. All sample data were subjected to normality and homogeneity of variance tests. Using analysis of variance to compare the means of multiple sample groups, and comparing the means of pairwise samples using the q-test method, namely Student Newman Kueuls (SNK) method. For comparisons involving multiple indicators (such as multiple blood routine and biochemical indicators before and after treatment), in order to control the risk of false positives caused by multiple comparisons, Bonferroni correction method was used to adjust the significance level of each test to *P* < 0.05/indicator number. The categorical variables were represented by percentages and their 95% confidence intervals. When the overall proportion p approaches 0 or 1, the interval was calculated using the Clopper Pearson method. Pearson’s chi square test or Fisher’s exact test was used to analyze differences between categorical variables.

It should be noted that this study was an exploratory study, and some subgroup analyses (such as comparisons between small sample drug combinations, changes in individual laboratory indicators, etc.) had not undergone multiple comparison correction. The relevant results should be considered exploratory, and their statistical significance should be interpreted with caution and further validated in subsequent studies. *P* < 0.05 indicates a statistically significant difference.

## Results

### Overview of the incidence of MPP in children

A total of 3395 cases of MPP were treated. There were 133 cases of refractory pneumonia, including 35 cases of combined bacterial infection, 6 cases of combined adenovirus infection, 7 cases of combined Chlamydia pneumoniae(CP) infection, 3 cases of MP induced thrombosis, and 82 cases of purity RMPP included in the experimental study. See Table 1.

**Table 1:** Overview of 133 cases of RMPP

Group	Number of cases	Percentage(95%CI)
Complex and refractory MPP	133/3395	3.92%(3.30%~4.64%)
MP combined with bacterial infection	35/3395	1.03%(0.72%~1.43%)
MP combined with adenovirus infection	6/3395	0.18%(0.07%~0.39%)
MP combined with CP infection	7/3395	0.21%(0.09%~0.43%)
MP causes thrombosis	3/3395	0.09%(0.02%~0.26%)
Purity RMPP	82/3395	2.43%(1.94%~3.01%)

### MP macrolide resistance test results

We conducted macrolide resistance molecular testing on 35 children with MP, and the results showed that 33 cases (94.3%) of the tested samples had clear A2063G resistance mutations. The remaining 2 cases (5.7%) did not detect any known drug-resistant mutations. Due to the small sample size of sensitive strains, effective statistical comparisons cannot be conducted. In RMPP, there was no significant difference in clinical characteristics between the drug-resistant detection group and the undetected group. The clinical feature comparison results are shown in Table 2.

**Table 2:** Comparison of clinical characteristics between the drug resistance testing group (Group 1) and the non-testing group (Group 2)

Variable	Group	*N*	Mean ± Sd	t	*P*
Age(year)	1	35	6.45 ± 2.21	1.97	0.05
	2	47	7.36 ± 1.96		
Weight(kg)	1	35	22.76 ± 7.35	1.91	0.06
	2	47	26.39 ± 9.32		
WBC(10^9^/L)	1	35	9.31 ± 4.32	0.83	0.40
	2	47	8.56 ± 3.81		
N(10^9^/L)	1	35	6.09 ± 3.58	0.05	0.96
	2	47	6.06 ± 3.07		
L(10^9^/L)	1	35	2.39 ± 1.39	0.79	0.43
	2	47	2.15 ± 1.33		
HB(g/L)	1	35	129.31 ± 10.62	1.69	0.09
	2	47	133.21 ± 10.10		
PLT(10^9^/L)	1	35	329.29 ± 120.29	0.69	0.49
	2	47	313.87 ± 96.20		
CRP(mg/L)	1	35	16.11 ± 18.89	0.78	0.44
	2	47	13.23 ± 14.46		
ALT(U/L)	1	35	28.74 ± 48.76	1.02	0.31
	2	47	64.85 ± 204.14		
AST(U/L)	1	35	34.63 ± 35.09	0.32	0.75
	2	47	37.66 ± 47.24		
UREA(mmol/L)	1	35	3.80 ± 0.96	0.44	0.66
	2	47	3.69 ± 1.24		
CREA(µmol/L)	1	35	35.40 ± 7.61	0.32	0.75
	2	47	34.77 ± 9.88		
LDH(U/L)	1	35	415.40 ± 220.51	0.45	0.65
	2	47	436.50 ± 193.61		
CK(U/L)	1	35	76.80 ± 65.33	0.94	0.35
	2	47	94.66 ± 97.03		
CK-MB(ng/mL)	1	35	3.45 ± 1.14	0.73	0.47
	2	47	3.26 ± 1.09		
ESR(mm/h)	1	35	31.63 ± 17.69	1.77	0.08
	2	47	25.55 ± 13.38		
D-D(mg/L)	1	35	1.38 ± 1.49	0.13	0.89
	2	47	1.42 ± 1.66		

ALT: Alanine aminotransferase; AST: Aspartate aminotransferase; CK: Creatine kinase; CK-MB: creatine kinase isoenzymes MB; CREA: Blood creatinine; CRP: C-reactive protein; D-D: D-dimer; ESR: erythrocyte sedimentation rate; HB: hemoglobin. L: lymphocyte; LDH: Lactate dehydrogenase; N: neutrophils; PLT: platelets; Urea: Blood urea; WBC: White blood cells

### Blood test results of RMPP patient on admission (before levofloxacin application)

There were 71 cases of RMPP patients with significantly elevated LDH, followed by 61 cases of elevated D-D, 56 cases of elevated ESR, and 54 cases of elevated C-reactive protein (CRP). Elevated liver enzymes was observed in 31 cases, and the impact of myocardial enzymes was relatively small, followed by HB with the smallest impact. LDH, D-D, ESR, CRP may have a significant correlation with the severity of RMPP. Please refer to Table 3 for specific information.

**Table 3:** Laboratory results of RMPP children on admission (before levofloxacin application)

Laboratory examination(Reference range )	Value/ (proportion/95%CI)	Laboratory examination(Reference range )	Value/ (proportion/95%CI)
**WBC**↑**or**↓**(4.4–11.9 × 10**^**9**^**/L)**	22/82(26.83%[17.03%~36.62%])	**ALT**↑**(0-42U/L)**	22/82(26.83%[17.03%~36.62%])
**N**↑**or**↓**(1.2-7.0 × 10**^**9**^**/L)**	23/82(28.05%[18.12%~37.98%])	**AST**↑**(0-59U/L)**	9/82(10.98%[04.07%~17.89%])
**PLT**↑**or**↓**(188–472 × 10**^**9**^**/L)**	18/82(21.95%[12.80%~31.10%])	**LDH**↑**(120-250U/L)**	71/82(86.58%[79.05%~94.12%])
**HB**↓**(112–149 g/L)**	3/82(3.66%[0.49%~9.37%])	**CK**↑**(40-200U/L)**	4/82(4.88%[ 0.91% to 10.80%]
**CRP**↑**(0-6 mg/L)**	54/82(65.85%[55.37%~76.34%])	**CK-MB**↑**(0-5ng/mL)**	6/82(07.31%[01.56%~13.07%])
**ESR**↑**(0.0–20 mm/h)**	56/82(68.29%[58.01%~78.58%])	**CREA**↑**(13–75µmol/L)**	11/82(13.41%[05.88%~20.95%])
**D-D**↑**(0.00-0.55 mg/L)**	61/82(74.39%[64.74%~84.04%])		

### Clinical efficacy of levofloxacin treatment

After the application of levofloxacin, the clinical symptoms of the child improved significantly, with an average fever reduction time of 1.96 ± 1.52 days and an average hospital stay of 12.04 ± 3.13 days. At discharge, all pleural effusion was absorbed, and most of the lobar changes in the lungs disappeared. After 4–12 weeks of outpatient follow-up, all lung lesions disappeared and were completely absorbed, with a cure rate of 100% (86.59% -100%, *N* = 82, Clopper Pearson method).

### Blood routine and biochemical test results before and after the application of levofloxacin

After treatment with levofloxacin, all test indicators in the blood routine showed no changes in WBC, N, PLT, etc.(*P* > 0.05) except for an increase in L value (*P* < 0.05). After the increase in L values, they are all within the normal range. There was no difference in ALT before and after treatment, AST decreased compared to before, indicating that there was no damage to liver function after the use of levofloxacin. LDH and CK-MB decreased after medication (*P* < 0.05), while CK remained unchanged. Blood CREA and blood UREA both decreased (*P* < 0.05) and there was no damage to renal function. See Table 4.

**Table 4:** Comparison of laboratory results of RMPP children before and after levofloxacin application $\left( {\bar {x}\, \pm \,{\mathrm{s}}} \right)$

Laboratory examination(Reference range )	Before levofloxacin application	After levofloxacin application	*P*
WBC(4.4–11.9 × 10^9^/L)	8.88 ± 4.03	9.99 ± 4.15	0.09
N(1.2-7.0 × 10^9^/L)	6.07 ± 3.27	5.94 ± 3.50	0.81
L(1.2–3.8 × 10^9^/L)	2.25 ± 1.35	3.35 ± 1.71	0.00
PLT(188–472 × 10^9^/L)	319.88 ± 106.75	340.79 ± 101.48	0.20
ALT(0-42U/L)	49.44 ± 158.07	22.67 ± 24.30	0.13
AST(0-59U/L)	36.37 ± 42.23	21.12 ± 7.42	0.00
LDH(120-250U/L)	427.29 ± 204.46	343.66 ± 105.57	0.00
CK(40-200U/L)	87.04 ± 84.95	65.85 ± 65.43	0.08
CK-MB(0-5ng/mL)	3.34 ± 1.11	2.74 ± 1.01	0.00
CREA(13–75µmol/L)	35.04 ± 8.93	30.24 ± 7.20	0.00
UREA(2.7−7.0mmol/L)	3.73 ± 1.13	3.13 ± 0.90	0.00

### Adverse reactions in children treated with levofloxacin

A total of 9 cases of adverse drug reactions occurred, accounting for 10.98%. Among them, rash was more common in 5 cases, and lower limb pain disappeared in 1 case upon discharge. No abnormal mental symptoms (excitement or irritability, depression or low mood) were observed. No serious adverse reactions were observed. No adverse reactions or sequelae were observed during the 12-month clinical follow-up. See Table 5.

**Table 5:** Adverse reaction manifestations of pediatric patients

Adverse reaction	Number of cases	Percentage
Abdominal discomfort	2	2.44%
Rash	5	6.10%
Headache and dizziness	1	1.22%
Lower limb pain	1	1.22%

## Discussion

Since the outbreak of the COVID-19 epidemic in 2019, the Chinese Mainland had implemented strict prevention and control measures, such as strictly restricting the flow of people, and wearing masks in public places, and so on. The prevention and control measures will not be lifted until 2022. In the following a year, from the second half of 2023 to 2024, there was a nationwide MP infection pandemic, and the incidence rate of MPP remained high. We had counted a total of 3573 cases of pneumonia admitted to the Children’s Respiratory Ward of Weifang People’s Hospital from November 2023 to December 2024, including 3395 cases of MP infection (or combined MP infection), accounting for about 95%. There were a total of 133 cases of RMPP, including 35 cases of combined bacterial infection, 6 cases of combined adenovirus infection, 7 cases of combined chlamydia infection, and 3 cases of MP induced thrombosis. 48 cases with concurrent infections caused by other pathogenic microorganisms were treated with other antibiotics in combination and 3 cases of children with concomitant thrombosis who were transferred for treatment that were not included in our study. Among them, 82 children with single MP infection had recurrent fever and cough. They were given intravenous erythromycin or azithromycin for more than 7 days, and were treated with methylprednisolone or even human immunoglobulin. However, the children’s body temperature did not decrease, coughing worsened, and even wheezing occurred, and there was progress in lung imaging, which was considered as RMPP. MP infection resistant to macrolide antibiotics may be one of the main causes of RMPP occurrence [6]. In addition, the occurrence of RMPP may also be related to the combination of drug-resistant MP and viral infections, especially adenovirus [8]. The 82 children with RMPP included in the study were all diagnosed with lobar pneumonia, including 18 cases with pleural effusion and 5 cases with respiratory distress. The youngest child is 1 year and 8 months old, and the oldest is 13 years and 10 months old. All children who did not respond well to treatment with macrolide antibiotics promptly adjusted their treatment plan and switched to intravenous injection of levofloxacin. The fever in the children gradually subsided, and the symptoms of cough and asthma in the children gradually improved. When the child’s temperature was normal, clinical condition had significantly improved, pulmonary imaging lesions had markedly absorbed or resolved, discharge criteria were met, and oral medication could be tolerated, levofloxacin was switched from intravenous injection to oral administration at an equivalent dose. The oral bioavailability of levofloxacin is approximately 99% (ranging from 95% to 100%). This means that when taking a pill orally, almost all of the active ingredients can be absorbed into the bloodstream, and the effect is almost the same as intravenous injection. Within the therapeutic dose range, there is a linear relationship between absorption and elimination. As the dose increases, the blood drug concentration increases proportionally. Administering the same dose of levofloxacin (e.g. 500 mg IV and 500 mg PO) can achieve almost the same blood drug concentration time curve (i.e. AUC) and peak concentration (Cmax) in the patient’s body. Therefore, when switching from intravenous injection to oral administration, there is no need to adjust the dosage, and a seamless transition can be achieved [9]. We used levofloxacin to treat RMPP in children, and the dosage and usage of the drug were strictly in accordance with the “Diagnosis and Treatment Guidelines for Mycoplasma Pneumonia in Children (2023 Edition)” issued by the National Health Commission of China. 10 mg/(kg/time) is given to children aged 6 months to 5 years old, q12h; Children aged 5–16 years old, 10 mg/(kg/time), qd, Intravenous injection or oral administration. To prevent or reduce the potential harm of levofloxacin, the course of treatment should be controlled within 10 days as much as possible. The shortest course of treatment was 5 days, and only one case had a longest course of treatment of 12 days. The average fever reduction time of levofloxacin application was 1.96 ± 1.52 days. The average course of treatment for levofloxacin was 7.44 ± 1.50 days. After follow-up, all patients were completely cured and all lobar changes disappeared completely.

Levofloxacin is a second-generation quinolone drug with broad-spectrum antibacterial activity and strong antibacterial activity. It has strong antibacterial activity against most Enterobacteriaceae bacteria such as Escherichia coli, Klebsiella, Proteobacter, Salmonella, Shigella, and Gram negative bacteria such as Haemophilus influenzae, Legionella pneumophila, and Neisseria gonorrhoeae. It also has antibacterial effects on gram-positive bacteria such as Staphylococcus aureus, Streptococcus pneumoniae, and Streptococcus pyogenes, as well as MP and CP, and is therefore widely used in adults. It is one of the most powerful weapons for treating bacterial infections [10]. However, animal experiments have shown that quinolone antibiotics may permanently damage the soft tissues of weight-bearing joints in young animals, and the safety of this product in infants, young children, and adolescents under 18 years old has not been determined. Therefore, it is not suitable for children and adolescents under the age of 18 [11]. China only approves levofloxacin for use in children aged 6 months to 18 years after inhalation anthrax exposure [12].

According to research on the application of levofloxacin in children outside of China, it was mainly used for the prevention of bacteremia in hematopoietic stem cell transplant patients before implantation, patients aged ≥ 6 months who received hematopoietic stem cell transplantation [13], and pediatric patients who received intensified chemotherapy for leukemia [14, 15]. In South Africa and India, levofloxacin could be used for preventive treatment of multidrug resistant tuberculosis children [16, 17]. However, with the increase of drug-resistant bacteria and the good antibacterial efficacy of levofloxacin, levofloxacin had begun to be used in children in China, but it was mostly used in severe infection cases, mainly for anti bacterial infections, and had good clinical effects. The side effects are also controllable and related to dosage and treatment duration [18].

Adverse reactions of levofloxacin are more common in adults, reported to be as high as 12% [19], mainly gastrointestinal reactions such as abdominal discomfort or pain, diarrhea, nausea or vomiting, loss of appetite, indigestion, etc. Secondly, it can lead to central nervous system reactions such as dizziness, headache, drowsiness, insomnia, and other related brain diseases [20], as well as induce various mental illnesses such as epileptic seizures, mental abnormalities, restlessness, confusion, hallucinations, tremors, etc [21]. Another adverse reactions is allergic reactions such as rash and itching on the skin. Joint pain is rare. Occasional occurrences include hematuria, phlebitis, and crystallized urine, which are more common when used at high doses [22]. A small number of patients may experience elevated serum ALT, elevated blood urea nitrogen, and decreased peripheral blood count (WBC), mostly mild and transient. There are rare reports of levofloxacin causing acute rhabdomyolysis [23], inducing myasthenia crisis [24], and leading to hyperpigmentation [25]. Fluoroquinolone drugs have multiple known adverse reactions, but few overdose events have been reported. Francesco M et al. [26] reported a case of a previously healthy middle-aged woman accidentally ingesting a dose of 7 g of levofloxacin. Afterwards, she suffered from hemiplegia due to ischemic stroke and went to the emergency department for treatment, taking tissue type plasminogen activator. Her brain imaging showed no ischemic damage and symptoms were relieved within 24 h. This case highlights the potential adverse reactions of acute overdose of levofloxacin.

The incidence of adverse reactions to levofloxacin observed in children was 10.98% in our study, lower than that in adults. Rash was the most common adverse reaction, occurring in 5 cases, followed by abdominal discomfort in 2 cases, dizziness in 1 case, and lower limb pain in 1 case. Except for administering oral antihistamines to children with rash for symptomatic treatment, no special interventions were provided for the others. Symptoms resolved spontaneously, and no long-term sequelae were identified. The case of lower limb pain occurred in a 6 years and 9 months old child. Bradley JS’s study indicates that the risk of cartilage damage caused by levofloxacin in pediatric patients is uncommon, with no clinical monitoring within five years or being reversible [27]. A meta-analysis conducted by Siyu Li found that the overall incidence of adverse drug events in children using systemic quinolones was 5.39%, which is lower than the incidence of adverse reactions in our study, it is not excluded that it is related to the extensive use of other drugs before the application of levofloxacin. The most common adverse drug reaction was gastrointestinal (with an incidence rate of 2.02%). Quinolone induced musculoskeletal adverse reactions in children are not common (0.76%) [28]. In our observations, rash was more common in children, and it cannot be ruled out that the children had used other drugs before taking levofloxacin. After treatment with levofloxacin in all children patients, there were no abnormalities in WBC, N, PLT values, liver and kidney function, or myocardial enzymes, and no serious adverse reactions were observed. There are reports that the risk of tendinitis caused by the use of fluoroquinolone drugs (FQ) in adults persists for up to three months after discontinuation of FQ(half of the cases occurred after FQ was discontinued). High risk factors include: age (> 65 years), associated (especially systemic), corticosteroid therapy, impaired kidney function (acute or chronic renal failure), duration of treatment, high cumulative dose, and transplant reception. These effects are explained by degenerative damage to collagen and reduced synthesis of proteoglycans [29]. We followed up 82 children for 1 year and did not find any serious adverse reactions or sequelae. We also did not observe any changes in the cartilage, tendons, or mental state of the children. Unfortunately, there is a lack of electrocardiogram data, and the data on the impact on children’s electrocardiogram and QTc is blank. In the future, research in this area should be strengthened and long-term follow-up should be conducted.

This study observed a very high rate of macrolide resistance (94.3%) in a subgroup of evaluable pediatric patients, which is highly consistent with multiple reports in China and East Asia in recent years that the resistance rate of Mycoplasma pneumoniae isolates in children is > 80%. This discovery provides a key etiology explanation for the “refractory” nature of the cases in this study: the clinical treatment failure of macrolide antibiotics is largely driven by the inherent resistance of the pathogen. Therefore, timely switching to non macrolide antibiotics (such as levofloxacin in this study) becomes a reasonable treatment strategy when initial macrolide therapy is ineffective. This study observed that levofloxacin still demonstrated excellent efficacy (100% clinical cure) in a population containing a high proportion of resistant strains, confirming its potential value in addressing macrolide resistance from a clinical perspective.

## Limitations of the research

This study is a real-world clinical observation study, and there have not been many cases of levofloxacin use in clinical institutions at all levels in Weifang City in the past. Therefore, comparative analysis cannot be conducted, and the inference of causal relationships may have corresponding confounding factors, including the therapeutic effect of previous drugs, the effect of other adjuvant therapy drugs, and the self limitation of disease conditions, which cannot be ruled out. When extrapolating the research results, the role of confounding factors needs to be fully considered. In this single-center prospective cohort, levofloxacin use in pediatric RMPP was associated with short-term clinical and laboratory improvements. Given the limited standardized resistance confirmation, lack of a comparator, and implementation details of safety surveillance, causal inference is limited. Confirmation in larger, comparative studies with protocolized long-term safety monitoring is warranted.

Another limitation of this study is that it is a single center study and lacks a randomized control group during the same period. Moreover, all patients came from the same tertiary hospital, which may represent more severe cases. Therefore, caution should be exercised when generalizing the results of this study to RMPP patients with milder conditions, younger or older ages, different geographical or medical resource backgrounds. Future multicenter, prospective, randomized controlled trials will help validate our findings.

## Conclusions

In recent years, levofloxacin has been increasingly used in children, from its initial application in leukemia and cancer patients, to anti-tuberculosis treatment, to the treatment of severely infected patients, and until now, we have used levofloxacin to treat RMPP, all of which have achieved good results. Moreover, levofloxacin has high safety and no serious adverse reactions have been found. At present, the medication instructions for levofloxacin are still prohibited for children under 18 years old, mainly due to concerns about joint and muscle disease in children. Based on our research and recent literature reports, there have been no reports of severe adverse reactions caused by levofloxacin in children. Balancing efficacy and safety, we believe that the use of levofloxacin in children is safe, at least for the short-term course of treatment. Of course, longer-term follow-up is necessary.

## Electronic supplementary material

Below is the link to the electronic supplementary material.

Supplementary Material 1

## Data Availability

The data that support the findings of this study are available from the author. Lexiang Yu upon reasonable request.
