# Supplementary material for: Clinical application and safety observation of levofloxacin in children with refractory Mycoplasma pneumoniae pneumonia
Source: BMC Infect Dis. 2026 Apr 29;26:1157. doi: 10.1186/s12879-026-13433-0 (PMC13274193; doi:10.1186/s12879-026-13433-0)
Supplement: Supplementary file 1 — Supplementary Material 1 [file 12879_2026_13433_MOESM1_ESM.doc]

| Test Item | Reagent/Instrument | Manufacturer |
| --- | --- | --- |
| Respiratory pathogen nucleic acid | 13 respiratory pathogen multiplex detection kits | Ningbo Haiershi Gene Technology Co., Ltd |
| Mycoplasma pneumoniae antibody | IFlash3000 chemiluminescence analyzer | Shenzhen Yahuilong Biotechnology Co., Ltd |
| Complete blood count | Fully automatic blood cell analyzer, BC-7500CRP | Shanghai Mindray Co., Ltd |
| Blood biochemistry | ADVIAXPTFully automatic biochemical analyzer | Siemens Germany |
| ESR (Erythrocyte Sedimentation Rate) | **Monitor-100Fully automatic erythrocyte sedimentation rate analyzer** | Siemens Germany |
| D-dimer | SysmexCS-5100/2500automated coagulation analyzer | Hisense Mikan from Japan |
|  |  |  |

1. **Experimental instruments and equipment**

**2.Electrocardiogram examination status before application of levofloxacin**

| Electrocardiogram examination status | N（%） |
| --- | --- |
| Normal electrocardiogram | 24（29.3） |
| Sinus arrhythmia (sinus arrhythmia, tachycardia or bradycardia) | 27（32.9） |
| T-wave change | 2（2.4） |
| Right deviation of electric axis | 2（2.4） |
| ST segment changes | 1（1.2） |
| Atrioventricular block | 2（2.4） |
| V1-V3R waves in chest leads  Incremental defects | 1（1.2） |
| No electrocardiogram was performed | 23（28.0） |
| Total | 82（100.0） |
